# Supplementary material for: Preparation of Amino-Functional UiO-66/PIMs Mixed Matrix Membranes with [bmim][Tf2N] as Regulator for Enhanced Gas Separation
Source: Membranes (Basel). 2021 Jan 4;11(1):35. doi: 10.3390/membranes11010035 (PMC7824137; doi:10.3390/membranes11010035)
Supplement: Supplementary file 1 [file membranes-11-00035-s001.pdf]

Supporting Information

Preparation of Amino-functional UiO-66/PIMs Mixed Matrix Membranes with [bmim][Tf_2N] as Regulator for Enhanced Gas Separation

Jiangfeng Lu [1], Xu Zhang [1], Lusheng Xu [1], Guoliang Zhang [1,*], Jiuhan Zheng [1], Zhaowei Tong [1], Chong Shen [2] and Qin Meng [2,*]

1 Center for Membrane and Water Science &Technology, Institute of Oceanic and Environmental Chemical Engineering, State Key Lab Breeding Base of Green Chemical Synthesis Technology, Zhejiang University of Technology, Hangzhou 310014, China; jln106412@sina.com (J.L.); xz669592@sina.com (X.Z.); xulusen@zjut.edu.cn (L.X.); zhengjiuhan@163.com (J.Z.); lzw199168@163.com (Z.T.)

2 Department of Chemical and Biological Engineering, State Key Laboratory of Chemical Engineering, Zhejiang University, Hangzhou 310027, China; rainbows@zju.edu.cn

* Correspondence: guoliangz@zjut.edu.cn (G.Z.); mengq@zju.edu.cn (Q.M.); Tel.: +86-571-8832-0863 (G.Z.); +86-571-8795-1227 (Q.M.)

1. Supplementary chart

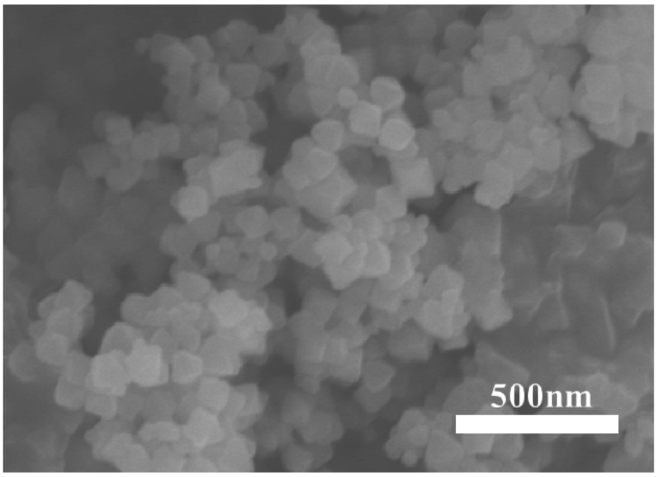

Figure S1. SEM image of UiO-66-NH_2 particles.

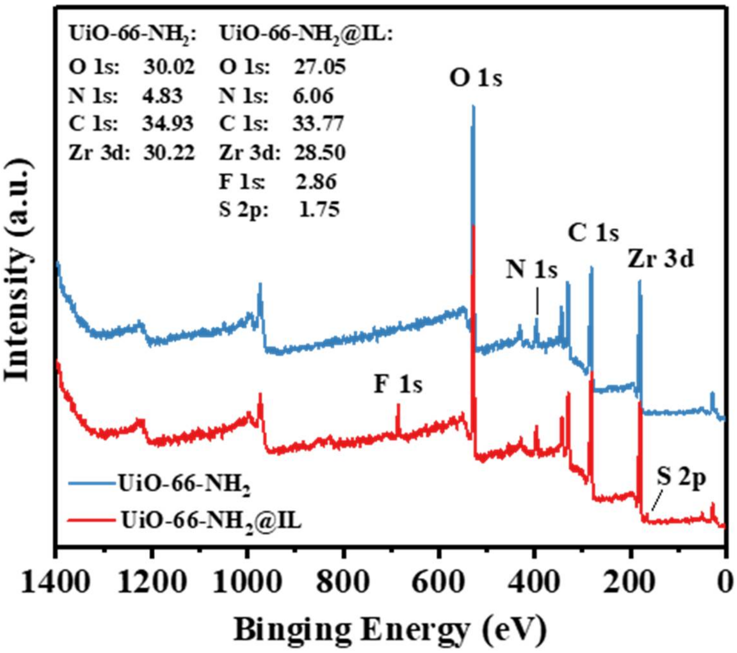

Figure S2. XPS data of UiO-66-NH_2 and UiO-66-NH_2@IL.

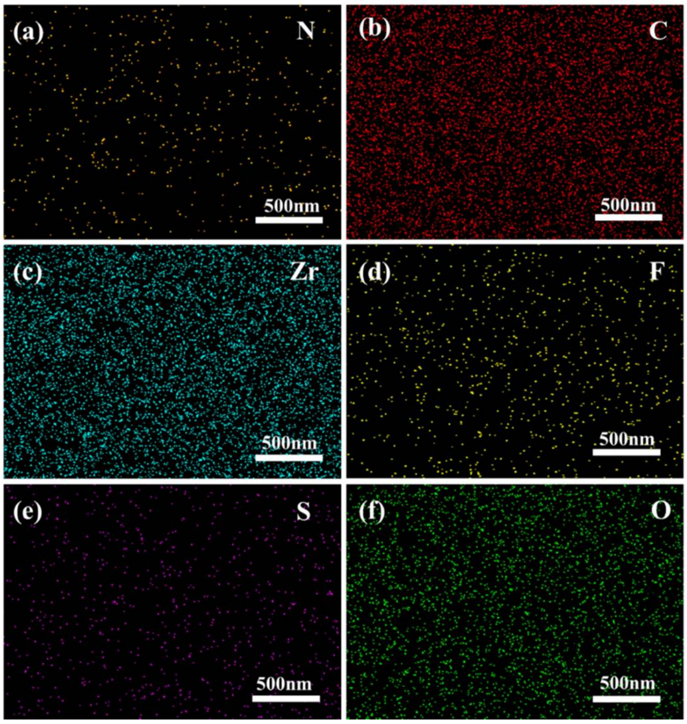

Figure S3. EDS mapping of the surface of UiO-66-NH_2@IL.
